# The value of ^68^Ga-PSMA enhanced PET-CT and MR-PET in patients with biochemical recurrent prostate cancer

**DOI:** 10.1186/1470-7330-15-S1-P39

**Published:** 2015-10-02

**Authors:** E Rummeny, K Holzapel, T Maurer, G Weirich, E Gschwend, M Eiber

**Affiliations:** 1 Klinikum rechts der Isar, Technical University Munich, Munich, Germany

## Aim of study

In patients with prostate cancer increased levels of PSMA can be measured. Recently a new tracer, ^68^Ga-PSMA, was developed as a specific marker for hybrid imaging (PET/CT, MR-PET). In this study we evaluated the accuracy of ^68^Ga-PSMA in patients with rising PSA after radical prosatectomy, so called “biochemical recurrent prostate cancer” (BRPC).

## Materials and methods

A total of 322 patients with BRPC underwent either a PET-CT or a MR-PET examination (Siemens Biograph mMR) after injection of about 150 mBq^68^Ga-PSMA. Images were evaluated in cosensus by one experienced nuclear medicine physician and one radiologist. Pelvine lymphnode dissection was performed in most of the patients according to a predefined template with 8 fields. Lymphnode involvement was evaluated according to a 5 point scale with a patient- and a field-based analysis. These findings were stratified according to PSA-values.

## Results

Four patients were excluded from the study for different reasons. Sensitivity for detection of recurrence was 95.7 % for PSA-values ≥ 2ng/ml, 81.4 % for PSA-values of 1-2 ng/ml, 76% for PSA-values 0.5-1 ng/ml, and 51% for PSA values ≤ 0.5 ng/ml. In comparison to the MR-images alone MR-PET was of superior diagnostic value.

## Conclusions

MR-PET using ^68^Ga-PSMA is a sensitive and highly accurate technique for the diagnosis of biochemical reccurence of prostate cancer after radical prostatectomy. It yields high diagnostic performance at relatively low PCA-values.

